# Prevalence and associated factors of insomnia symptoms among pregnant women in the third trimester in a moderately developing region of China

**DOI:** 10.1186/s12889-023-17269-0

**Published:** 2023-11-23

**Authors:** Xu Chen, Yuan Liu, Meilin Liu, Fanli Min, Jiao Tong, Wei Wei, Lizhou Sun, Teng Zhang, Qian Meng

**Affiliations:** 1 Lianyungang Maternal and Child Health Hospital, 669 Qindongmen Street, Haizhou District, Lianyungang, Jiangsu China; 2 First Affiliated Hospital of Nanjing Medical University, Nanjing, Jiangsu China

**Keywords:** Insomnia symptoms, Pregnant women, Associated factors, In the third trimester, China

## Abstract

**Background:**

Insomnia is the most common sleep disorder in the general population, especially among pregnant women, and it is considered a major public health issue. Not only can it cause mental and physical problems in pregnant women, but it may also affect the growth of the fetus. However, there are few reports on the prevalence and influencing factors of insomnia symptoms in third-trimester women in China. The objective of this study was to assess the prevalence of insomnia symptoms among pregnant women in the third trimester in a moderately developing region of China and to further explore the associated factors of insomnia symptoms from various aspects.

**Methods:**

A cross-sectional survey was conducted among eligible pregnant women in the third trimester from December 2022 to February 2023. Data on socio-demographic characteristics, clinical characteristics, and behavioral and psychological characteristics of pregnant women were collected through a structured questionnaire. The Chi-square test and multivariate logistics regression were applied to explore the associated factors of insomnia symptoms.

**Results:**

A total of 535 pregnant women in the third trimester were included in this study, and the prevalence of insomnia symptoms was 59.8%. Multivariate logistic regression analysis revealed that pregnant women who lived together with elders (*OR*: 0.58, 95% *CI*: 0.40–0.86), had low perceived stress (*OR*: 0.58, 95% *CI*: 0.35–0.97), had no threatened abortion (*OR*: 0.55, 95% *CI*: 0.32–0.93) and had good doctor-patient communication (*OR*: 0.66, 95% *CI*: 0.45–0.98) were more likely to stay away from insomnia symptoms. However, pregnant women with anxiety symptoms (*OR*: 2.27, 95% *CI*: 1.28–4.03), fear of childbirth (*OR*: 1.63, 95% *CI*: 1.11–2.40) and a high experience of COVID-19 fear (*OR*: 1.61, 95% *CI*: 1.03–2.54) tended to have insomnia symptoms.

**Conclusions:**

The prevalence of insomnia symptoms in pregnant women is high in Lianyungang city in eastern China in the third trimester. Insomnia symptoms is influenced by multiple factors. There is an urgent need to develop interventions to reduce the prevalence of insomnia symptoms in the third trimester and to focus on pregnant women with risk factors for insomnia symptoms.

## Background

Sleep is a key factor affecting people’s quality of life. Good sleep is conducive to physical health, and sleep quality determines the quality of life [1]. Pregnant women’s hormonal changes, psychological changes, and behavioral changes may affect their sleep [2]. The incidence of insomnia is high in pregnant women, with at least one third of pregnant women reported to suffer from insomnia, and it predominates in the second and third trimesters [3]. Several research results from different countries and regions showed that the probability of insomnia during pregnancy was about 18.5–61.6% [4–7]. Two meta-analyses of 24,362 participants reported that the overall risk of sleep problems in pregnant women was 38.2% and the overall prevalence of third-trimester insomnia was 42.4% [8, 9]. A study from Turkey reported that the prevalence of insomnia among 486 pregnant women was 52.2%, and the risk of insomnia in the third trimester was 2.03 times that of the first and second trimesters [10], and the risk of sleep problems in the third trimester was increased [6, 11].

Pregnant women suffering from insomnia may have significant damage in emotions, physical functions and other aspects [12, 13]. If the insomnia symptoms of pregnant women are not timely interfered with, the physical and mental health of pregnant women will be damaged, and the pregnancy outcome of pregnant women and newborns will be significantly adversely affected [14, 15]. The results of a meta-analysis indicated that insomnia and poor sleep quality increase the odds of suicidal risk in pregnant women by more than threefold [16]. Other studies have found a longitudinal relationship between sleep quality and maternal related quality of life during pregnancy, and sleep difficulties are one of the determinants of pregnant women’s related quality of life [17, 18]. These studies all suggest a causal relationship between insomnia and prognosis. A recent study from the United States found that pregnant women with insomnia had a higher cumulative incidence of obstetric comorbidities [19]. Researchers have shown that sleep disturbances at the end of pregnancy are associated with higher rates of high blood pressure, preeclampsia, and postpartum depression [20]. The available evidence suggests a clear association between sleep disturbances during pregnancy and gestational diabetes [21].

In previous studies, insomnia symptoms were related to a variety of social, psychological, and biological mechanisms [2, 22–24]. In terms of socio-demographic characteristics, the associated factors of insomnia mainly include the mother’s age, education level, marital status, employment, perceived stress and so on [25–27]. In terms of clinical symptoms, the associated factors of insomnia mainly include gestational age, parity, gestational hypertension, pregnancy symptoms and so on [27, 28]. In terms of behavioral psychological characteristics, the associated factors of insomnia mainly include partner’s smoking habits, physical exercise, anxiety symptoms, fear of childbirth, experience of COVID-19 fear and so on [13, 28–30]. Considering the differences in racial, economic, cultural, social structure, and other aspects between the local region and the regions studied above, there may also be differences in insomnia symptoms and associated factors. Therefore, it is necessary to analyze these associated factors of insomnia symptoms in pregnant women in the local region. It is worth noting that some other factors may also be associated with insomnia symptoms, such as living conditions, residential noise, threatened abortion, and doctor-patient communication. Residential noise is one of the important factors affecting insomnia [31, 32], but it has not received sufficient attention among pregnant women. Living conditions, threatened miscarriage, and doctor-patient communication can cause a series of psychological problems during pregnancy, such as anxiety and depression [33–35], and may also potentially affect insomnia in pregnant women. Therefore, it is also necessary to analyze the correlation between these factors and insomnia symptoms in pregnant women.

In recent years, most studies on insomnia among pregnant women focus on the whole pregnancy cycle. However, as the period with the highest incidence of insomnia, the relevant prevalence and associated factors have not been fully studied [36, 37]. In addition, several studies reported insomnia among pregnant women in some countries and regions, but few studies reported in a moderately developing region of China. Due to the COVID-19 pandemic, pregnant women in the third trimester of pregnancy are at increased risk of experiencing physical or psychological discomfort due to illness [30]. Therefore, the aim of this study is to analyze the prevalence of insomnia symptoms among pregnant women in the third trimester in a moderately developing region of China during the COVID-19 pandemic, and to explore the factors associated with insomnia symptoms in terms of socio-demographic characteristics, clinical characteristics, and behavioral and psychological characteristics. In order to reduce the insomnia rate of pregnant women in the third trimester, the implementation of targeted intervention measures to provide a theoretical basis.

## Materials and methods

### Study design and participants

A cross-sectional survey was conducted from December 2022 to February 2023 at the Lianyungang Maternal and Child Health Hospital, the only national third-grade class-A maternal and child health hospital in Lianyungang, Jiangsu Province, Eastern China. During this period, pregnant women in the third trimester who met the inclusion criteria and received perinatal services in the outpatient department of Lianyungang Maternal and Child Health Hospital were mobilized to complete a paper-based questionnaire. The inclusion criteria included (1) age ≥ 18 years; (2) gestational period of ≥ 28 weeks; (3) no intellectual disability or severe disease; (4) ability to understand the study protocol and agree to participate in the study. The data collectors in this study were a fixed survey team composed of five people who had uniform training. Before giving out the questionnaires, the survey team members introduced the purpose, content, confidentiality, benefits, and risks of the study to the pregnant women in detail. The minimum sample size required for this study was calculated using the single population proportion formula. Due to the lack of relevant studies at the study site, to obtain the maximum sample size, we used a prevalence of insomnia symptoms as 50% (*P* = 50%), 95% *CI*, a margin error of 5%, and a rate of non-response of 20%. The sample size required was calculated to be 461. To obtain more reliable conclusions, a total of 550 pregnant women in the third trimester were recruited, of which 15 pregnant women did not complete the questionnaire due to time constraints and were excluded. Finally, 535 pregnant women in the third trimester with complete questionnaires without missing items were included in the study, resulting in a participation rate of 97.3%.

### Measurements

A structured questionnaire was used to collect insomnia symptoms, socio-demographic characteristics, clinical characteristics, and behavioral and psychological characteristics of pregnant women in the third trimester. The questionnaire was developed based on a review of a large body of relevant literature and consultation with experts in the relevant field, and was pre-surveyed at the study site and revised. Socio-demographic characteristics included age, education level, marital status, employment, residence, living situation, perceived poor physical resistance, perceived stress, and residential noise. Clinical characteristics included assisted pregnancy (referring to the use of external forces to achieve the purpose of successful pregnancy, including artificial fertilization and test-tube babies), parity, history of cesarean section, threatened abortion, gestational hypertension, and pregnancy symptoms. Pregnancy symptoms included nausea, lower abdominal pain, loss of appetite, and other symptoms since pregnancy. Behavioral and psychological characteristics included the partner’s smoking habits, partner’s drinking habits, time of electronic screen exposure per day, physical exercise, doctor-patient communication, anxiety symptoms, fear of childbirth, and experience of COVID-19 fear.

Insomnia symptoms were assessed using the Chinese version of the Insomnia Severity Index (ISI), a widely used tool to assess the severity of clinical insomnia symptoms [4, 38]. ISI is often used to assess the subjective sleep quality of pregnant women and has been shown to have good reliability and validity [4, 38–40]. It consists of seven items, each rated on a 5-point Likert scale ranging from 0 to 4. Total score ranges from 0 to 28, with high score indicating severe insomnia symptoms. A cutoff value of 8 or higher indicates the presence of insomnia symptoms [15]. ISI is widely used in epidemiological investigations [40]. In the current study, its Cronbach’s alpha value was 0.897.

Doctor-patient communication was assessed using a component derived from the Consumer Assessment of Healthcare Providers and Systems (CAHPS) [41]. This component has been shown to have good reliability and validity [42]. It consists of four items, which involve careful listening, the way of explanation, the degree of respect, and time of service delivery by medical staff. Each item was scored on a scale from 1 (never) to 4 (always). The total score is based on the sum of four items and ranges from 4 to 16. A higher total score indicates better doctor-patient communication [43]. In most scales without recommended cut-off values, the average value was used as the cut-off point [44, 45]. In this study, the mean value of the scale was 12.9, and the median score was 13.0. The grouping results of both as cut-off points were the same. Therefore, pregnant women in the third trimester with a total score of average or above in this study were labeled as having good doctor-patient communication. In the current study, it had a good internal consistency (Cronbach’s alpha value = 0.885).

Anxiety symptoms were measured using the Generalized Anxiety Disorder-7 (GAD-7) scale, which is easy to understand and can be completed quickly [46]. GAD-7 has been shown to have good reliability and validity in pregnant women [40, 47]. It consists of seven items, each rated on a 4-point Likert scale ranging from 0 (not at all) to 3 (nearly every day). Item scores were summed to obtain a total score, which ranged from 0 to 21. The higher the total score is, the more severe the anxiety symptoms. A total score of 7 or greater was defined as the presence of anxiety symptoms [48]. In the current study, its Cronbach’s alpha value was 0.934.

Fear of childbirth was assessed using the Chinese version of the Childbirth Attitudes Questionnaire (CAQ) [49]. The Chinese version of CAQ has been validated in Chinese pregnant women with good internal consistency [50]. It consists of 16 items, each rated on a 4-point Likert scale ranging from 1 (never) to 4 (high). The sum of item scores is the total score of the questionnaire, which ranges from 16 to 64. High scores reflect high levels of fear of childbirth. A total score greater than or equal to 28 was considered the presence of fear of childbirth [49]. It demonstrated excellent internal consistency in the current study (Cronbach’s alpha value = 0.951).

Experience of COVID-19 fear was assessed using the Fear of COVID-19 Scale (FCV-19 S), which measures emotional fears occurring during the COVID-19 pandemic [51]. FCV-19 S has been verified to have good reliability and validity in many countries [52, 53]. It consists of seven items, each rated on a 5-point Likert scale ranging from 1 (strongly disagree) to 5 (strongly agree). Item scores were summarized as the total score of the scale, which ranged from 7 to 35. The higher the total score is, the higher the level of fear. A total score greater than 21 was defined as having high fear [52]. In the current study, it had a good internal consistency (Cronbach’s alpha value = 0.892).

### Statistical analyses

Questionnaires without missing items and logical errors were entered into a database established by Epidata3.1 (EpiData Association, Odense, Denmark) software by double entry. All statistical analyses were performed using SPSS21.0 (IBM Corporation, Armonk, State of New York) software. Continuous variables are presented as the mean and standard deviation (SD) and categorical variables as frequency and percentage. Considering the sample size and the number of variables, it was chosen in the current study to first screen the independent variables by univariate analysis to ensure that the appropriate variables entered the multivariate logistic regression model. Univariate analysis was performed using the Chi-square test to explore the differences in insomnia symptoms between different groups. Variables with significant differences in Chi-square test analysis were included in the multivariate logistic regression model to explore the independent effect of each variable on insomnia symptoms after controlling for confounding factors. Collinearity tests were performed to assess the collinearity between predictor variables before performing the multivariate logistic regression analysis. The test showed that the variance inflation factor (VIF) of each predictor variable was less than 10, and the tolerance was much greater than 0.1. This indicates the absence of collinearity. To gain insight into the association between predictors and insomnia symptoms, Shapley Additive Explanations (SHAP) summary plots were used. Statistical significance was taken as *P* < 0.05 (two-tailed).

## Results

### Socio-demographic characteristics and their relationship with insomnia symptoms

A total of 535 pregnant women in the third trimester with no missing data were included in the study, with a mean age of 29.4 ± 4.5 years. A small number of pregnant women (18.9%) had a secondary school education level or below, and a large number (94.2%) were satisfied with their current marital status. More than half of pregnant women (53.8%) were currently employed and 56.6% were living with elders. A small number of pregnant women (4.9%) perceived that they had poor physical resistance, and more than one in five (23.9%) thought that current stress was high. Almost one in ten pregnant women (10.3%) live in an area exposed to noise. Among the participants, 320 (59.8%) pregnant women in the third trimester had insomnia symptoms, and the mean score of insomnia symptoms was 8.9 ± 5.0. Univariate analysis indicated that marital status, living situation, perceived poor resistance, perceived stress, and residential noise were significantly associated with insomnia symptoms (*P* < 0.05) (Table 1).

**Table 1:** Prevalence of insomnia symptoms according to socio-demographic characteristics

Variable	Total n (%)	Insomnia symptoms n (%)		*P*
		No	Yes	
Age (years)				
< 35	468(87.5)	187(40.0)	281(60.0)	0.775
≥35	67(12.5)	28(41.8)	39(58.2)	
Education level				
Secondary or below	101(18.9)	45(44.6)	56(55.4)	0.604
High school	78(14.6)	30(38.5)	48(61.5)	
University or above	356(66.5)	140(39.3)	216(60.7)	
Marital status				
Satisfied	504(94.2)	209(41.5)	295(58.5)	**0.015**
Unsatisfied	31(5.8)	6(19.4)	25(80.6)	
Employment				
Yes	288(53.8)	117(40.6)	171(59.4)	0.823
No	247(46.2)	98(39.7)	149(60.3)	
Residence				
Urban	357(66.7)	137(38.4)	220(61.6)	0.226
Rural	178(33.3)	78(43.8)	100(56.2)	
Living situation				
Living only with husband	232(43.4)	79(34.1)	153(65.9)	**0.011**
Live together with elders	303(56.6)	136(44.9)	167(55.1)	
Perceived poor physical resistance				
Agree	26(4.9)	3(11.5)	23(88.5)	**0.002**
Disagree	509(95.1)	212(41.7)	297(58.3)	
Perceived stress				
Low	407(76.1)	185(45.5)	222(54.5)	**< 0.001**
High	128(23.9)	30(23.4)	98(76.6)	
Residential noise				
Yes	55(10.3)	13(23.6)	42(76.4)	**0.008**
No	480(89.7)	202(42.1)	278(57.9)	

**Notes**: Significant values are in bold

### Clinical characteristics and their relationship with insomnia symptoms

Among 535 participants, 6.5% of pregnant women conceived with an assisted pregnancy, and more than half (55.7%) were multiparous. About one in five pregnant women (19.4%) had a previous cesarean section, and 18.9% had threatened abortion. A large proportion of pregnant women (85.0%) had experienced pregnancy symptoms such as pregnancy vomiting and lower abdominal pain since pregnancy. Univariate analysis showed that threatened abortion and pregnancy symptoms were significantly associated with insomnia symptoms (*P* < 0.05) (Table 2).

**Table 2:** Prevalence of insomnia symptoms according to clinical characteristics

Variable	Total n (%)	Insomnia symptoms n (%)		*P*
		No	Yes	
Assisted pregnancy				
Yes	35(6.5)	11(31.4)	24(68.6)	0.274
No	500(93.5)	204(40.8)	296(59.2)	
Parity				
Primiparity	237(44.3)	96(40.5)	141(59.5)	0.893
Multiparity	298(55.7)	119(39.9)	179(60.1)	
History of cesarean section				
Yes	104(19.4)	39(37.5)	65(62.5)	0.533
No	431(80.6)	176(40.8)	255(59.2)	
Threatened abortion				
Yes	101(18.9)	27(26.7)	74(73.3)	**0.002**
No	434(81.1)	188(43.3)	246(56.7)	
Gestational hypertension				
Yes	19(3.6)	7(36.8)	12(63.2)	0.762
No	516(96.4)	208(40.3)	308(59.7)	
Pregnancy symptoms				
Yes	455(85.0)	171(37.6)	284(62.4)	**0.003**
No	80(15.0)	44(55.0)	36(45.0)	

**Notes**: Significant values are in bold

### Behavioral and psychological characteristics and their relationship with insomnia symptoms

Among the study subjects, more than half of the pregnant women (52.7%) were exposed to electronic screens for more than 5 h per day, and 44.5% were never physically exercised. Almost half of the pregnant women (51.6%) had good doctor-patient communication, and 20.2% had anxiety symptoms. A large number of pregnant women (56.6%) had fear of childbirth, and more than a quarter (27.7%) had a high experience of COVID-19 fear. Univariate analysis found that doctor-patient communication, anxiety symptoms, fear of childbirth, and experience of COVID-19 fear were significantly associated with insomnia symptoms (*P* < 0.05) (Table 3).

**Table 3:** Prevalence of insomnia symptoms according to behavioral and psychological characteristics

Variable	Total n (%)	Insomnia symptoms n (%)		*P*
		No	Yes	
Partner’s smoking habits				
Yes	209(39.1)	81(38.8)	128(61.2)	0.589
No	326(60.9)	134(41.1)	192(58.9)	
Partner’s drinking habits				
Yes	167(31.2)	66(39.5)	101(60.5)	0.832
No	368(68.8)	149(40.5)	219(59.5)	
Time of electronic screen exposure per day (hours)				
≤ 5	253(47.3)	102(40.3)	151(59.7)	0.954
>5	282(52.7)	113(40.1)	169(59.9)	
Physical exercise				
Often	58(10.8)	28(48.3)	30(51.7)	0.341
Sometimes	239(44.7)	97(40.6)	142(59.4)	
Never	238(44.5)	90(37.8)	148(62.2)	
Doctor-patient communication				
Good	276(51.6)	123(44.6)	153(55.4)	**0.033**
Poor	259(48.4)	92(35.5)	167(64.5)	
Anxiety symptoms				
Yes	108(20.2)	20(18.5)	88(81.5)	**< 0.001**
No	427(79.8)	195(45.7)	232(54.3)	
Fear of childbirth				
Yes	303(56.6)	96(31.7)	207(68.3)	**< 0.001**
No	232(43.4)	119(51.3)	113(48.7)	
Experience of COVID-19 fear				
Low	387(72.3)	175(45.2)	212(54.8)	**< 0.001**
High	148(27.7)	40(27.0)	108(73.0)	

**Notes**: COVID-19: corona virus disease 2019; Significant values are in bold

### Associated factors of insomnia symptoms

**Fig. 1:**
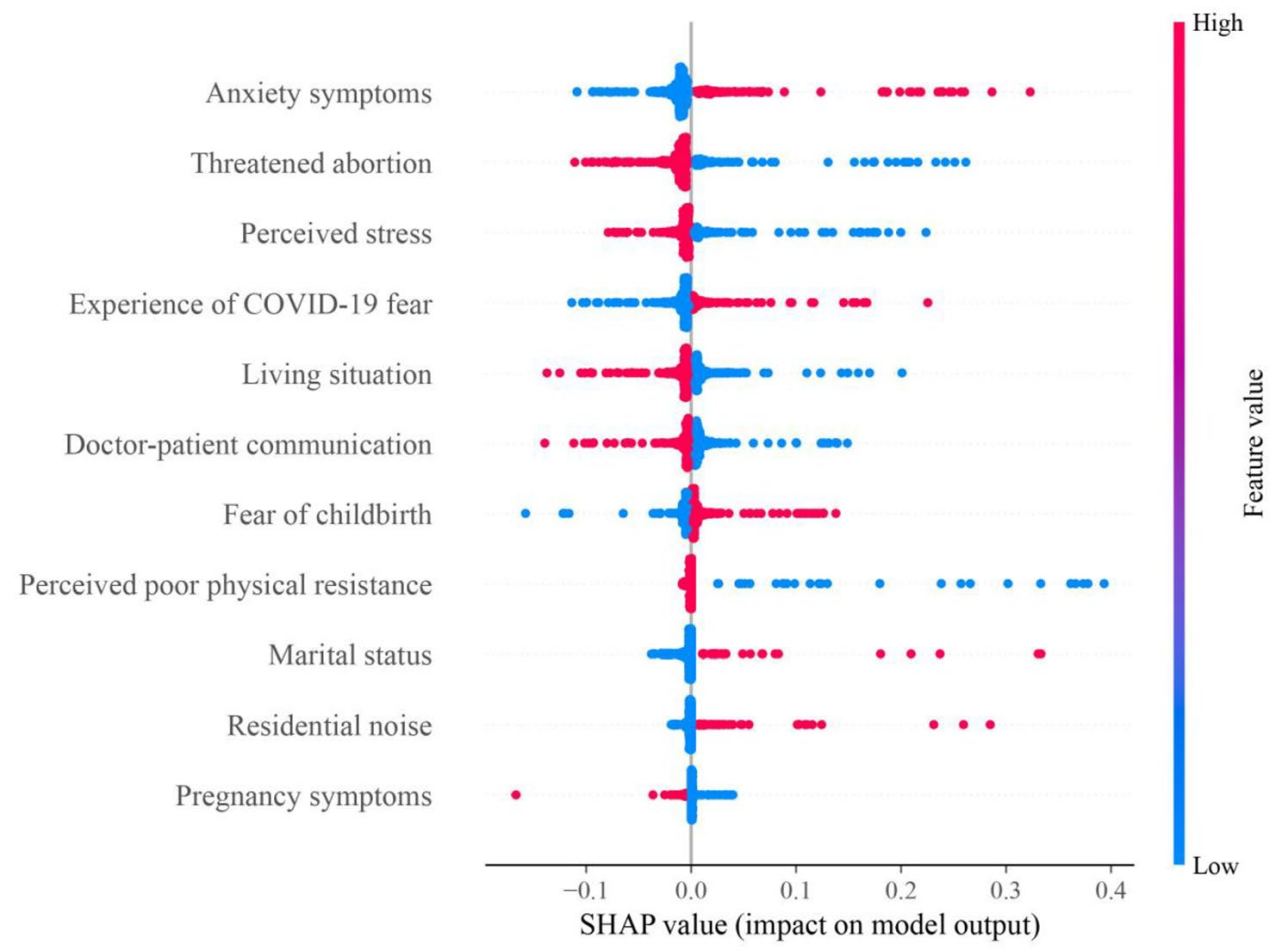
SHAP summary plot. The higher the SHAP value of a feature, the higher the probability of the presence of insomnia symptoms. Red represents higher feature values, and blue represents lower feature values; COVID-19: corona virus disease 2019; SHAP: Shapley Additive Explanations

Multivariate logistic regression analysis revealed that pregnant women who lived together with elders (*OR*: 0.58, 95% *CI*: 0.40–0.86), had low perceived stress (*OR*: 0.58, 95% *CI*: 0.35–0.97), had no threatened abortion (*OR*: 0.55, 95% *CI*: 0.32–0.93) and had good doctor-patient communication (*OR*: 0.66, 95% *CI*: 0.45–0.98) were more likely to stay away from insomnia symptoms. However, pregnant women with anxiety symptoms (*OR*: 2.27, 95% *CI*: 1.28–4.03), fear of childbirth (*OR*: 1.63, 95% *CI*: 1.11–2.40) and a high experience of COVID-19 fear (*OR*: 1.61, 95% *CI*: 1.03–2.54) were more likely to have insomnia symptoms (Table 4). Figure 1 shows the relationships between the 11 features and SHAP values. Anxiety symptoms showed the most positive impact on model output (Fig. 1).

**Table 4:** Multivariate logistic regression analysis of factors associated with insomnia symptoms

Variable	*OR*	95% *CI*		*P*
		Low	High	
Marital status				
Satisfied	1			
Unsatisfied	1.92	0.72	5.12	0.195
Living situation				
Living only with husband	1			
Living together with elders	0.58	0.40	0.86	**0.006**
Perceived poor physical resistance				
Agree	1			
Disagree	0.39	0.11	1.41	0.149
Perceived stress				
Low	0.58	0.35	0.97	**0.037**
High	1			
Residential noise				
Yes	1.61	0.80	3.24	0.184
No	1			
Threatened abortion				
Yes	1			
No	0.55	0.32	0.93	**0.025**
Pregnancy symptoms				
Yes	1			
No	0.63	0.38	1.06	0.082
Doctor-patient communication				
Good	0.66	0.45	0.98	**0.037**
Poor	1			
Anxiety symptoms				
Yes	2.27	1.28	4.03	**0.005**
No	1			
Fear of childbirth				
Yes	1.63	1.11	2.40	**0.013**
No	1			
Experience of COVID-19 fear				
Low	1			
High	1.61	1.03	2.54	**0.039**

**Notes**: COVID-19: corona virus disease 2019; Significant values are in bold

## Discussion

To our knowledge, this study fills the gap in the prevalence and associated factors of insomnia symptoms among pregnant women in a moderately developing region of China in the third trimester. Our data showed that the prevalence of insomnia symptoms was 59.8% in the third trimester of pregnancy. Data from a recent study in China using the same measurements and assessment criteria as this study showed that the prevalence of insomnia symptoms among pregnant women was 18.5% in the central cities of northern China (Beijing), central China (Wuhan), and western China (Lanzhou) [4]. Several previous studies reported the prevalence of insomnia symptoms among pregnant women in the third trimester abroad: 19.8% in Australia [7], 61.6% in Norway [54], 39.6% in Poland [6], and 33.2% in the USA [55]. The prevalence varies by country and region, which may be related to differences in lifestyle, social structure, and measurement methods. The results of this study suggest that insomnia symptoms are highly prevalent among pregnant women and is an important public health problem that needs to be given adequate attention in a moderately developing region of China in the third trimester. Moreover, this study revealed that living situation, perceived stress, threatened abortion, doctor-patient communication, anxiety symptoms, fear of childbirth, and experience of COVID-19 fear were associated factors for insomnia symptoms among pregnant women in the third trimester.

In socio-demographic characteristics, our data indicated that both living situation and perceived stress were associated with insomnia symptoms among pregnant women in the third trimester. Previous studies showed that inadequate family support was associated with insomnia symptoms in pregnant women [56]. However, there are few reported correlations between living situation and insomnia symptoms among pregnant women in the third trimester. Our data showed that pregnant women living together with elders were more likely to stay away from insomnia symptoms than that living only with husband in the third trimester. This may be attributed to the irreplaceable support provided by elders for pregnant women, such as care, encouragement, and sharing of pregnancy and childbirth-related experiences [57]. However, for these, the husband might not be available or might not be doing enough. Our data also showed that perceived stress was an associated factor for insomnia symptoms among pregnant women in the third trimester, which was similar to the findings of several previous studies [26, 58]. Actually, most people face enormous living pressure due to high rent or mortgage loans in moderately developing regions of China [59]. When faced with the high costs of feeding, medical care, and education that their children would have to endure in the future, their quality of life was even worse, and their lived more stressful [60]. These perceived stressors might contribute to insomnia symptoms among pregnant women. Notably, a good relationship with a partner contributed to low levels of perceived stress [61].

In clinical characteristics, our data indicated that threatened abortion was associated with insomnia symptoms among pregnant women in the third trimester. Previous studies showed that about a quarter of all threatened abortion would progress to a full abortion in the following weeks [62]. Furthermore, in subsequent pregnancies, women who experienced the threatened abortion were at increased risk for adverse pregnancy outcomes, including pregnancy loss, antepartum hemorrhage, preterm birth, perinatal death, and low birth weight infants [63, 64]. In addition, women with threatened abortion could suffer from several psychological problems, such as anxiety and depression [65, 66]. These potential threats prompted pregnant women to develop insomnia symptoms. Therefore, healthcare providers should provide quality medical care for women with threatened abortion to reduce their insomnia symptoms rate during pregnancy [66].

In behavioral and psychological characteristics, our data indicated that doctor-patient communication, anxiety symptoms, fear of childbirth, and experience of COVID-19 fear were associated with insomnia symptoms among pregnant women in the third trimester. Previous studies showed that good patient-doctor communication was positively associated with patient sleep quality [67, 68]. This study obtained similar results, indicating that doctor-patient communication was associated with insomnia symptoms among pregnant women in the third trimester. In fact, good patient-doctor communication shows enough patience and respect for the patient, which keeps the patient in a good mood and allows the patient to have a good quality sleep [67]. Our data showed that maternal anxiety symptoms were associated with insomnia symptoms in the third trimester of pregnancy, which was consistent with previous studies [5, 11]. Certain neurotransmitters, such as dopamine and 5-hydroxytryptamine, play a crucial role in regulating circadian rhythms and influence the quality of sleep [24, 69]. Anxiety could cause changes in these neurotransmitters, which in turn contribute to insomnia symptoms [24, 69]. Our data showed that fear of childbirth was associated with insomnia symptoms among pregnant women in the third trimester. Previous studies showed that pregnant women often obtain information about labor through various sources (e.g. doctors, the Internet, etc.) to be adequately prepared physically and mentally [70, 71]. However, this information, part of which was false or overblown, put a psychological burden on them as they face the impending birth and may further produce insomnia symptoms [72]. In addition, Our data showed that experience of COVID-19 fear was associated with insomnia symptoms in pregnant women, which was consistent with previous findings [30, 73]. This might be related to (1) fear of infection and transmission to the fetus, (2) cancellation of check-ups and pre-school classes, and (3) the inability to have close friends or partners during labor or the first few days after delivery, as well as family tensions [73]. Due to these factors, pregnant women might be under high levels of mental stress and experience further symptoms of insomnia [73].

Although this study provided some valuable findings, some limitations need to be elaborated. Firstly, this study was a cross-sectional study and causal relationships between variables could not be obtained, therefore further longitudinal studies are needed. Secondly, this study was only conducted in the third trimester of pregnancy and future studies need to include comparative analysis to clarify the differences in prevalence and predictors of the first and second trimester of pregnancy. Thirdly, this study only analyzed the subjective ISI scale and did not analyze the objective indicators, which might have caused some bias. In addition, this study was conducted in one region only, and it was difficult to represent other regions with different socio-economic and cultural backgrounds due to the influence of the socio-economic and cultural backgrounds.

## Conclusion

In summary, the results of this study indicate that the prevalence of insomnia symptoms was 59.8% among pregnant women in the third trimester. The results found that living situation, perceived stress, threatened abortion, doctor-patient communication, anxiety symptoms, fear of childbirth, and experience of COVID-19 fear were significant associated factors for insomnia symptoms among pregnant women in the third trimester. Considering the high prevalence of insomnia symptoms, there is an urgent need to develop interventions to reduce the prevalence of insomnia symptoms and to focus on its risk factors in the third trimester.

## Data Availability

The datasets generated and/or analyzed during the current study are not publicly available for ethical reasons but are available from the corresponding author upon reasonable request.

## Abbreviations

CAHPS: Consumer Assessment of Healthcare Providers and Systems
CAQ: Childbirth Attitudes Questionnaire
CI: Confidence interval
COVID-19: Corona virus disease 2019
GAD-7: Generalized Anxiety Disorder-7
ISI: Insomnia Severity Index
OR: Odds ratio
SD: Standard deviation
SHAP: Shapley Additive Explanations
VIF: Variance inflation factor
